# Significance and Predictive Value of Delphian Lymph Node in Papillary Thyroid Carcinoma

**DOI:** 10.2174/0115734056360214250215155608

**Published:** 2025-03-13

**Authors:** Yaqi Cui, Yimeng Li, Xinlu Yin, Jiadong Wang

**Affiliations:** 1 Department of Otorhinolaryngology, the Sixth People’s Hospital, Shanghai Jiao Tong University, Shanghai, China; 2 School of Clinical Medicine, YangZhou University, No. 136, Jiangyang Middle Road, Ganjiang District, Yangzhou City, Jiangsu Province, China; 3 Department of Head and Neck Surgery, Renji Hospital of Shanghai Jiao Tong University, School of Medicine, Pujian Road 160, 200001 Shanghai, China

**Keywords:** Delphian lymph node, papillary thyroid carcinoma, lymph node metastasis, predictive value, lateral lymph node

## Abstract

**Background:**

Delphian lymph node (DLN) metastasis is common in papillary thyroid cancer (PTC). However, few studies have specifically investigated the clinicopathologic characteristics of DLN metastasis in PTC. This study aimed to examine the incidence, risk factors, and predictive value of DLN in papillary thyroid carcinoma.

**Methods:**

In the present study, the clinicopathologic features and metastatic risks were statistically analyzed by reviewing 1837 patients with papillary thyroid carcinoma who underwent initial surgery in our department between January, 2022 and July, 2024.

**Results:**

Among the 1837 patients included in the study, DLN was detected in 925 patients (50.3%), of which 409 patients (22.3%) had confirmed DLN metastasis. In univariate analysis, DLN metastasis was correlated with age (≥55 years), bilateral cancer, multifocality, tumor location (isthmus cancer), central lymph node metastasis (CLNM), and lateral lymph node metastasis (LLNM). However, it was not correlated with gender distribution, tumor size, thyroiditis, thyroid-stimulating hormone (TSH) level, and BRAF mutation. Multivariate analysis showed that CLNM (p=0.03), LLNM (p=0.025), bilateral cancer, and tumor location (p=0.012) were independent risk factors for DLN metastasis. DLN involvement was mildly predictive of CLNM (sensitivity=29.76%, specificity=87.06%, positive predictive values=74.08%, negative predictive values=49. 93%, positive likelihood ratio=2.30, negative likelihood ratio=0.81) and moderately predictive of LLNM (sensitivity=49.36%, specificity=85.01%, positive predictive values=46.94%, negative predictive values=86.20%, positive likelihood ratio=3.29, negative likelihood ratio=0.60).

**Conclusion:**

Bilateral cancer, CLNM, LLNM, and isthmus cancer were independent risk factors for DLN metastasis. DLN metastasis could be used as a predictor for central and lateral lymph node metastasis. Positive DLN should be a warning signal to carefully evaluate central and lateral lymph nodes during thyroidectomy.

## 1 INTRODUCTION

Thyroid cancer is the most common endocrine malignancy, and its worldwide incidence has increased rapidly over the past few decades. In China, it has become the sixth most common malignant tumor in the female population (according to the Annual Reports of Cancer in China). Its incidence rate is increasing, from less than 1.78/100 000 in 1988 to nearly 90/100 000 in 2015. The most common type of thyroid cancer is papillary thyroid carcinoma (PTC), which accounts for approximately 85% of all thyroid cancers [1]. While PTC is known for its slow progression, lymph node metastasis is common, especially in the neck area. Regional lymph node metastasis develops in 20-90% of patients with PTC, with the central cervical compartment, or level VI node, being the primary zone of lymphatic involvement [2].

The Delphian lymph node (DLN), located on the fascia above the thyroid isthmus, lies between the cricoid and the thyroid cartilage. Despite being the first lymph node encountered and easily resected during thyroidectomy, the role of DLN in thyroid cancer patients is still controversial due to insufficient data [3]. While one large study suggests that DLN involvement is an early sign of thyroid cancer, others have argued that DLN involvement is misleading and unreliable. Moreover, the clinicopathologic risk factors previously reported to be associated with DLN metastasis are inconsistent, and details about the clinical and predictive values of DLN differ from published research [4, 5].

Therefore, this study aimed to observe the incidence, risk factors, and predictive values of DLN metastasis in PTC in order to improve the surgical method of thyroid cancer and reduce postoperative recurrence and metastasis. By reviewing 1837 patients with PTC who underwent their first operation in our department between January, 2022 and July, 2024, we found that DLN was detected in 925 patients (50.3%), and 409 patients (22.3%) had confirmed DLN metastasis. We also identified several independent risk factors of DLN metastasis, including bilateral cancer, central lymph node metastasis (CLNM), lateral lymph node metastasis (LLNM), and isthmus cancer. DLN involvement was found to be mildly predictive of CLNM and moderately predictive of LLNM. Our study provides new insights into the role of DLN in PTC and highlights the importance of careful evaluation of central and lateral lymph nodes in thyroidectomy for patients with positive DLN.

## 2 METHOD

### 2.1 Study Design

This was a retrospective study, and we reviewed the medical records of 1837 patients who underwent thyroid surgery from January, 2022 to July, 2024, in the Department of Head and Neck Surgery Renji Hospital, Shanghai Jiaotong University. All patients were diagnosed using high-resolution ultrasonography, and most of them were confirmed by fine needle aspiration (FNA).

#### 2.1.1 Criteria for Surgical Patients

The criteria for selecting surgical patients were as follows:

1. Patient with preoperative FNA report of papillary carcinoma (including cytologically confirmed papillary carcinoma or positive BRAF gene),

2. Strong suspicion of malignancy on preoperative ultrasound and no willingness of the patient to undergo FNA (at least Ti-Rads 4a and with microcalcifications, aspect ratio >1, poorly defined borders, and irregular morphology).

#### 2.1.2 Inclusion Criteria for this Study

The inclusion criteria for this study were as follows:

1. Postoperative diagnosis of papillary thyroid cancer, confirmed by pathology,

2. Age: Adult (>18 years),

3. All patients underwent thyroid surgery for the first time, with no preoperative indication of distant metastasis, and the surgical modalities included lobectomy of the thyroid gland, CLNM, and LLNM,

4. Follow-up data: Complete follow-up data, including recurrence, metastasis, and survival, and

5. Patients were required to sign an informed consent form and were willing to participate in the study.

#### 2.1.3 Exclusion Criteria for this Study

The exclusion criteria for this study were as follows:

1. Other types of thyroid cancer: *e.g*., follicular thyroid cancer, undifferentiated carcinoma, *etc*.,

2. Comorbidities with other malignant tumors: patients with a history of malignant tumors elsewhere were excluded,

3. Serious comorbidities: *e.g*., cardiac disease, liver or renal insufficiency, *etc*., which may affect treatment and prognosis,

4. Recent radiotherapy or chemotherapy: patients who have received radiotherapy or chemotherapy may affect the results of the study, and

5. Pregnant or lactating patients.

The DLN was removed as long as the operating surgeon was able to identify and resect an obvious nodule. Patients with no detectable DLN were considered negative for DLN metastasis. The relationship between DLN metastasis and sex, age, tumor size, bilateral or unilateral cancer, multifocality, thyroiditis, preoperative thyroid stimulating hormone (TSH) level, BRAFV600E mutation, central lymph node metastasis (CLNM), lateral lymph node metastasis (LLNM), and tumor location (isthmus or lobe) was recorded. This study was approved by the Ethics Committee of Renji Hospital, and written informed consent for surgical treatment was obtained from all patients, which is available for review at the medical records department of the hospital.

### 2.2 Statistical Analysis

Statistical analysis was performed with SPSS software version 26.0. Univariate analysis was performed using the Chi-square criterion, and multivariate analysis was carried out using logistic regression analysis. Variables found to be significantly different between groups in the univariate analysis were included in the multivariate logistic regression analysis. Differences were considered significant if P < 0.05.

## 3 RESULTS

### 3.1 CT Image Showing DLN

The DLN is frequently encountered during laryngeal or thyroid surgery. It is a lymphatic tissue located anterior to the larynx or cricoid cartilage, typically consisting of one or more lymph nodes located on the surface of the cricoid and cricothyroid cartilage. These nodes are positioned within the thyroid groove, between the cricoid and cricothyroid cartilages, the cricothyroid membrane, and the fascia on the surface of the cricothyroid cartilage. The number of DLNs is variable and usually consists of 1 to 4 round lymph nodes of varying sizes that receive lymph from the thyroglossal duct, larynx, and hypopharyngeal region. Due to the laryngeal cartilage, DLNs do not visualize well on ultrasound, so we chose CT to identify the location of DLNs. As shown in Fig. (**1**), DLN (indicated by the red arrow) was diagnosed as metastatic papillary thyroid carcinoma in two patients.

#### 3.1.1 Univariate and Multivariate Analyses of Risk Factors of DLN(+) and DLN(-) Patients

All patients in the study underwent total thyroidectomy and prophylactic central lymph node dissection. Among 1837 patients with PTC enrolled in the study, DLN was detected in 925 patients (50.3%), and among them, 409 (22.3%) had confirmed DLN metastasis. The mean numbers of detected and metastatic DLNs were 1.83 and 1.32, respectively, as mentioned in Table **1**. The patients without DLN were considered negative for metastasis (Table **1**). The mean age of these patients was 46.5 years, and the male-to-female ratio was 1:2.84.

As presented in Table **2**, there was no statistically significant difference observed between DLN(+) and DLN(-) patients in terms of sex distribution, tumor size, thyroiditis, preoperative TSH level, and BRAF mutation. Age (≥55 years or <55 years), bilateral or unilateral cancer, multifocality, CLNM, LLNM, and tumor location (isthmus or lobe) were significantly different between the two groups. Specifically, patients with DLN(+) were more likely to be older (≥55 years), have bilateral cancer, multifocality, CLNM, LLNM, and have tumors located in the isthmus. These variables were included in the multivariate logistic regression analysis.

When performing multivariate analyses, significant differences were found in the following factors: bilateral cancer, CLNM, LLNM, and tumor location (isthmus or lobe). However, there were no differences in age and multifocality between DLN(+) and DLN(-) groups (Table **3**).

#### 3.1.2 Predictive Value of DLN in CLNM and LLNM

Next, we investigated whether DLN metastasis was predictive of CLNM and LLNM. Due to its positional advantage of being the first lymph node seen intraoperatively, the predictive value of the DLN is very high. As presented in Table **4**, DLN involvement was moderately predictive of CLNM (sensitivity=29.76%, specificity=87.06%, positive predictive values=74.08%, negative predictive values =49.93%, positive likelihood ratio=2.30, negative likelihood ratio=0.81) and mildly predictive of LLNM (sensitivity=49.36%, specificity=85.01%, positive predictive values=46.94%, negative predictive values =86.20%, positive likelihood ratio=3.29, negative likelihood ratio=0.60). Patients with DLN metastasis were 2.3 times more likely to have central node disease and 3.29 times more likely to have lateral node disease compared to those without DLN metastasis. These data are summarized in Table **4**.

Of the 192 patients who underwent lateral neck dissection, the most frequently involved level was level III (46.88%), followed by level II (33.33%) and level VI (19.79%) (Table **5**).

## 4 DISCUSSION

In our present study, the frequency of DLN metastasis in PTC patients was 22.3% (409 of 1837). To the best of our knowledge, the metastatic rate of DLN involvement ranged from 8.2% to 36.2%, according to previous studies [4, 6-8]. Interestingly, the metastasis rate of DLN in PTC appears to be higher than that in laryngeal carcinoma, which was reported to be 4.6%, 6.5%, and 7.5% in recent studies [9-11]. However, it should be noted that laryngeal cancer is generally more aggressive, with a higher recurrence rate compared to PTC. Therefore, investigating DLN metastasis in PTC patients is essential and valuable.

The term “Delphian node” (DN) refers to the pre-laryngeal or pre-cricoid nodal tissue often identified during laryngeal or thyroid surgery. DLN is a component of the central lymph node (level VI). Its significance in total thyroidectomy lies in the fact that it is the initial lymph node encountered during the surgical procedure [12]. The DLN holds particular surgical significance as it is predominantly located on the surface and the first lymph node encountered during total thyroidectomy.

Several risk factors for DLN metastasis in PTC have been identified, including larger tumor size, the presence of extrathyroidal extension, lymph vascular invasion, further lymph node metastasis, and tumor location. Multivariate analysis was also performed in some of these studies. However, there is no consensus on accurately correlated factors of DLN metastasis, as some studies reported conflicting results. Previous studies reported that tumor size was not an independent risk factor in DLN metastasis in PTC [13-16], and some research works reported that multifocality was not associated with DLN metastasis [14-17]. In this retrospective study, we evaluated the clinical significance and predictive factors of DLN metastasis in 1837 patients. Results showed that DLN metastasis was correlated with bilateral cancer, CLNM, LLNM, and tumor location (isthmus or lobe) but not correlated with age, multifocality, sex distribution, tumor size, thyroiditis, preoperative TSH level, and BRAF mutation.

The DLN receives afferent lymphatic drainage from the upper poles of the thyroid gland, isthmus, and pyramidal lobe, as well as the epiglottis, aryepiglottic fold, and infraglottic region. Afferent lymph flows from the larynx and the thyroid gland into the DLN before the central and lateral lymph nodes, making it the first lymph node basin draining the thyroid gland. Based on DLN anatomy, DLN metastasis is expected to be closely correlated with the location of the lesions, particularly in the thyroid isthmus/upper third of the thyroid gland. Chai *et al.* analyzed 370 patients and reported that tumor location in the isthmus/upper third was an independent risk factor for DLN metastasis [16]. However, Gong *et al.* revealed that the difference in the location of the lesions was not significant (including the left and right and superior and inferior) in DLN metastasis [18], which is consistent with the research conducted by Zheng *et al.* [14]. Additionally, PTCs located in the isthmus were more likely to involve the DLN than PTCs located in other sites [12, 19]. Our present study demonstrated that carcinoma located in the isthmus is more likely to metastasize to DLN. Two reasons may account for the heterogeneity in the findings: First, the sample size is different in these studies, and most of them are single-center clinical studies. Second, the thyroid gland has abundant lymphangion, and DLN drains lymph from not only the upper thyroid gland and isthmus but also other areas [8, 20]. In conclusion, when preoperative findings suggest tumor location in the isthmus of the thyroid, surgeons should carefully evaluate the status of the DLN metastasis.

Furthermore, DLN positivity was found to be a strong predictor of central and lateral node metastasis. Our study showed that DLN-positive patients were 2.3 times more likely to have central node metastasis and 3.29 times more likely to have lateral node involvement, as presented in Table **4**. Other studies have also reported similar findings. For instance, Iyer *et al.* found that DLN-positive patients were approximately 5 times more likely to have further central node metastasis and 3.5 times more likely to harbor lateral node metastasis than DLN-negative patients [13]. Isaacs *et al.* reported that DLN-positive patients were 8 times more likely to have metastasis to the central compartment and 4 times more likely to have lateral neck metastases [4]. They also reported that probabilities of central cervical lymph node metastasis and lateral neck lymph node metastasis in the presence of DLN metastasis in level VI lymph nodes reached 85% and 83%, respectively. In fact, DLN is the most accurate predictor of lateral node disease and some researchers even suggested that DLN metastasis should be upstaged to N1b. Given these findings, some experts recommend that bilateral central neck lymph node dissection for DLN-positive patients, as contralateral paratracheal LNM among DLN-positive patients, is significantly higher than that among DLN-negative patients (41.9% vs. 9.6%, P < 0.001) regardless of tumor size [15].

Although DLN is not the most involved site among level VI lymph nodes in PTC [21], our data and the results from other studies confirm the crucial role of DLN in predicting central and lateral node metastasis. It is possible that although DLN may be affected later, it exhibits more aggressive behavior compared to other lymph node groups. Therefore, a positive DLN should serve as an alert signal to carefully evaluate and warrant greater attention to central and lateral lymph nodes. In fact, Zhou *et al.* recommended that if the DLN is positive on the frozen section, central lymph node dissection should be carefully considered even in clinically node-negative PTC to minimize the risk of regional recurrence and improve survival [22]. Regarding the extent of lymph node dissection, it is suggested that contralateral paratracheal lymph nodes should be dissected even in patients with unilateral carcinoma who have positive DLN freezing results. DLN (+) can be considered an independent risk factor for contralateral paratracheal lymph node metastasis in patients with unilateral PTC.

However, there is no consensus on whether prophylactic central lymph node dissection (especially the contralateral side) is essential if DLN is confirmed positive intraoperatively, and further multi-institutional studies are needed to address this issue.

As presented in Table **5**, in patients with DLN (+) and LLNM (+), level III is the most likely lymph node to metastasize. Lymph nodes in different areas drain lymph fluid from different locations. Previous studies confirmed that after cervical lymph node dissection in patients with PTC and lateral cervical lymph node metastasis, level III is the most prone to metastasis in the lateral cervical region [23]. After lateral lymph node dissection for patients with postoperative recurrence of PTC, it was observed that the most mobile region of the lateral neck was level III. Tumors located in the middle and upper part are more likely to have skip lateral region metastasis, and skip lateral region metastasis is also most likely to metastasize to level III lymph nodes [24, 25]. DLN, like level III of lateral lymph nodes, mainly absorbs lymph nodes in the middle and upper part of the thyroid gland. The wide range of lymphatic drainage and the rich and complex reflux system in level III may be the reason why patients with DLN metastasis are more likely to develop lymph node metastasis in level III.

DLN metastasis is an important prognostic factor in PTC and other cancers in the head and neck, which has a significant impact on the choice of treatment strategy and the prognosis of patients. Although our study suggests that DLN is important for predicting CLN and LLN, there is no clear guideline or expert consensus that suggests that the lateral neck lymph nodes should be enlarged after DLN metastasis. Only some scholars have suggested that the ipsilateral VI lymph nodes and the contralateral VI lymph nodes should be carefully and prophylactically enlarged in patients with DLN metastasis from unilateral thyroid cancer. We believe that as more attention is paid to DLN metastasis, more studies need to be conducted to reach a consensus on expanding the scope of dissection for patients with DLN metastasis. Moreover, there will be many new research directions in the future due to the special location and clinical significance of DLN. First, identifying biomarkers associated with DLN will allow the study of new imaging and biomarkers to improve the diagnostic accuracy of DLN metastasis. This will further enable the possibility of DLN metastasis to be identified preoperatively and improve clinicians' attention to DLN clearance during surgery and close follow-up of patients with DLN metastasis after surgery. Second, the impact of different surgical treatment modalities on the prognosis of patients with DLN metastasis was evaluated, providing a basis for individualized treatment. It also provides new indications and a basis for targeted drug therapy for patients with locally advanced disease. Finally, the study of the tumor microenvironment and its relationship with DLN lymph node metastasis may provide a basis for new therapeutic targets.

The article has some limitations; firstly, it is limited to a single-center study. Secondly, it lacks data on long-term follow-up. Thirdly, in the classification of tumor location, only the isthmus cancer and glandular lobe cancer are simply distinguished, and the upper, middle, and lower locations of the tumor are not refined. If the proportion of upper and middle cancers is high, the DLN metastasis rate and the central lymph node metastasis rate may be affected. Lastly, the data may be biased due to the fact that the source of the data is related to the surgical patients, while the information on the non-surgical patients is unknown.

## CONCLUSION

In summary, DLN metastasis was found to be associated with various clinical-pathologic factors. Therefore, DLN should be dissected in all patients with thyroid cancer. DLN also appears to be an adverse prognostic marker, and the presence of DLN metastasis should alert the surgeon to closely monitor the patient during follow-up to prevent the likelihood of advanced disease.

## Figures and Tables

**Fig. (1):**
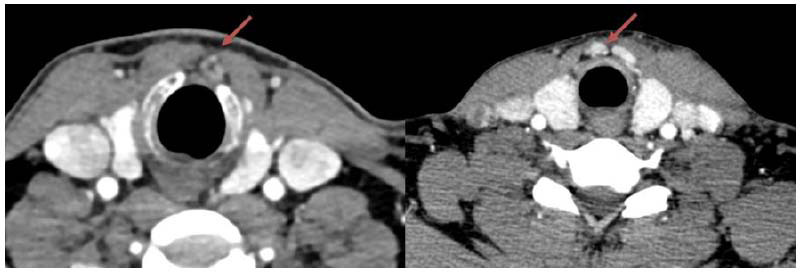
CT scan showing DLN in two patients, indicated by the red arrows.

**Table 1:** Rate of detection and metastasis of DLN (n=1837).

Detection of DLN	50.3% (925/1837)	
Metastasis of DLN	22.3% (409/1837)	
Mean no. of DLN detection	1.83	-
Mean no. of DLN metastases	1.32	-

**Table 2:** Demographic and clinicopathologic characteristics between DLN (+)/(-) patients:Univariate analysis.

**Variables**	-	**DLN(+) (n=409)**	**DLN(-) (n=1428)**	**χ^2^**	P-value*
Sex	-	-	-	0.225	0.635
-	Male	86	285	-	-
-	Female	323	1143	-	-
Age	-	-	-	7.752	0.005
-	≥55y	105	470	-	-
-	<55y	304	958	-	-
Tumor size		-	-	1.183	0.277
-	≥1.0cm	194	634	-	-
-	<1.0cm	215	794	-	-
Bilateral cancer		-	-	18.488	0.000
-	Yes	138	520	-	-
-	No	391	908	-	-
Mulifocality		-	-	6.927	0.008
-	Yes	73	182	-	-
-	No	336	1246	-	-
Thyroiditis		-	-	0.608	0.435
-	Yes	74	235	-	-
-	No	335	1193	-	-
Preoperative TSH level			-	1.189	0.275
-	<2.5	231	763	-	-
-	≥2.5	178	665	-	-
BRAF	-	-	-	0.816	0.665
-	Mutant	215	717	-	-
-	Wild type	15	50	-	-
-	Undetected	179	661	-	-
CLNM*	-	-	-	74.204	0.000
-	Positive	303	715	-	-
-	negative	106	713	-	-
LLNM	-	-	-	217.924	0.000
-	Positive	192	197	-	-
-	negative	217	1231	-	-
Location	-	-	-	25.991	0.000
-	Lobe	307	1224	-	-
-	Isthmus	102	204	-	-

* represent non-DLN central lymph node metastasis. CLNM: Central lymph node metastasis. LLNM: Lateral lymph node metastasis.

**Table 3:** Multivariate logistic regression analysis of DLN metastasis.

	**Exp (B)**	**P-value**	**95% CI**
Age (<55y)	3.794	0.068	0.908-15.85
Bilateral cancer	1.579	0.012	0.324-3.859
Mulifocality	3.857	0.083	2.118-7.024
CLNM	4.459	0.03	1.159-17.164
LLNM	4.5	0.025	1.207-16.773
Isthmus	4.937	0.017	1.331-18.311

**Table 4:** The ability of DLN metastasis to predict CLNM and LLNM.

	**Sn**	**Sp**	**PPV**	**NPV**	**LR+**	**LR-**
CLNM	29.76%	87.06%	74.08%	49.93%	2.30	0.81
LLNM	49.36%	85.01%	46.94%	86.20%	3.29	0.60

**Table 5:** Metastasis pattern in 192 patients with DLN and lateral neck metastasis.

Level II	64	33.33%	(64/192)
Level III	90	46.88%	(90/192)
Level IV	38	19.79%	(38/192)

## Data Availability

The data and supportive information are available within the article.

## LIST OF ABBREVIATIONS

DLN: Delphian Lymph Node
PTC: Papillary Thyroid Cancer
CLNM: Central Lymph Node Metastasis
LLNM: Lateral Lymph Node Metastasis
TSH: Thyroid-stimulating Hormone
FNA: Fine Needle Aspiration
DN: Delphian Node

## STANDARDS OF REPORTING

STROBE guidelines were followed.
